# Exploring the Relationship Between Game Performance and Physical Demands in Youth Male Basketball Players

**DOI:** 10.3390/jfmk10030293

**Published:** 2025-07-29

**Authors:** Javier Espasa-Labrador, Carlos Martínez-Rubio, Franc García, Azahara Fort-Vanmeergaehe, Jordi Guarch, Julio Calleja-González

**Affiliations:** 1 INEFC-Barcelona Research Group on Sport Sciences (GRCE), National Institute of Physical Education of Catalonia (INEFC), University of Barcelona (UB), 08038 Barcelona, Spain; javierespasa@roadtoperformance.com; 2 Physical Preparation and Load Monitoring Department, Football Club Barcelona, 08028 Barcelona, Spain; 3 Barça Innovation Hub, Football Club Barcelona, 08028 Barcelona, Spain; 4 Road to Performance Center, 15007 Coruña, Spain; 5 Department of Education, Faculty of Education Sciences, University of Almeria, 04120 Almería, Spain; carloscmrubio@gmail.com; 6 SPORT Research Group (CTS-1024), CIBIS, Research Center, University of Almería, 04120 Almería, Spain; 7 FPCEE Blanquerna, SAFE Research Group, Ramon Llull University, 08022 Barcelona, Spain; azaharafv@blanquerna.url.edu; 8 Segle XXI Female Basketball Team, Catalan Federation of Basketball, 08950 Esplugues de Llobregat, Spain; 9 NTT Data, 08005 Barcelona, Spain; jgua2418@gmail.com; 10 Department of Physical Education and Sport, Faculty of Education and Sport, University of the Basque Country (EHU), 01007 Vitoria, Spain; 11 Faculty of Kinesiology, University of Zagreb, 10110 Zagreb, Croatia

**Keywords:** external load, load monitoring, performance, injury prevention

## Abstract

**Background:** Understanding the relationship between physical demands and game performance is essential to optimize player development and management in basketball. This study aimed to examine the association between game performance and physical demands in youth male basketball players. **Methods:** Fifteen players (16.3 ± 0.7 years) from a Spanish 4th division team were monitored over seven official games. Game performance variables were extracted from official statistics, including traditional and advanced metrics. Physical demands were monitored using an Electronic Performance Tracking System device, combining a positioning system and inertial sensors. Partial correlations, controlling for minutes played, were calculated to explore associations between physical demands and performance variables, both for the entire team and by playing position. **Results:** Significant correlations between physical demands and game performance were observed. Points scored correlated strongly with total distance and high-intensity accelerations, while assists correlated with high-intensity decelerations. Inertial metrics, such as player load and the number of jumps, showed large correlations with points, two-point attempts, and the efficiency rating. Positional analysis revealed stronger and more numerous correlations for centers compared to guards and forwards. Inertial sensor-derived metrics exhibited a greater number and strength of correlations than positioning metrics. **Conclusions**: Game performance and physical demands are intrinsically related, with specific patterns varying by playing position. Inertial sensors provide valuable complementary information to positioning systems for assessing physical demands in basketball. These findings can assist practitioners in tailoring monitoring and training strategies to optimize performance and manage player workload effectively.

## 1. Introduction

Basketball is one of the most widely played and followed sports globally, engaging millions of participants and enthusiasts [1]. Its popularity stems from sport’s dynamic nature, physical demands, technical skills, and tactical complexity [2]. In addition, it is characterized by high-intensity actions such as changes of direction, jumps, and physical impacts, which require a minimum level of physical fitness, combined with the mastery of specific technical skills (e.g., shooting, passing, dribbling) and tactical knowledge (e.g., reading concepts, game systems, strategies) and the ability to make quick decisions in a chaotic and ever-changing environment [3].

This combination of elements necessitates the active participation of all players on the court during both offensive and defensive phases. As a result, recording and analyzing game events has become a key aspect of performance evaluations [4]. Over the past decades, these methods have evolved from traditional statistics to advanced metrics, with the National Basketball Association serving as a benchmark in the identification, formulation, and promotion of performance evaluation metrics [5]. These statistics have played a crucial role in assessing both individual and team performance. Traditionally, the most used metrics have focused on basic parameters such as points, rebounds, and assists. However, with the advent of technological tools and the incorporation of data science methodologies, advanced statistics have emerged, enabling deeper and more precise evaluations [4]. These advanced metrics integrate temporal variables and probabilities of events occurring, such as the number of potential rebounds available following missed shots. By relativizing events, new metrics have been developed. Among these variables, the offensive rating, defensive rating, and usage rate, have been highlighted as the most used, offering a more holistic understanding of a player’s contribution to team success. These metrics surpass traditional measures like player efficiency ratings, which aggregate various variables [5].

Concurrently, interest in assessing the physical demands faced by players during competition has grown as well. These demands include variables such as total distance covered, the number and intensity of sprints, and high-intensity accelerations and decelerations during games. These metrics are derived from a change in positioning of the players over time. A method and metrics were imported to indoor sports from outdoor sports, such as football and rugby [6]. Outdoor sports demand high rates of locomotion, in which both success and fatigue-related actions are linked to a change of position. In the case of indoor sports, where spaces for each player are smaller, there are not so many possibilities of displacement, and the performance in the game has been related more to high-intensity actions in different planes of movement, including acceleration, changes of direction, jumps, or impacts [7]. Many of these actions occur with little or no movement of the players in space, so positioning systems, especially in vertical jumping actions or fighting between players, have limitations in monitoring physical demands in basketball [8]. For this reason, the use of inertial sensors (a combination of accelerometers, gyroscopes, and magnetometers) has been proposed for the quantification of physical demand to assess physical metrics, allowing the recording of actions that occur with little or no displacement and at a high speed, thus avoiding the limitations of positioning systems [8,9]. It also allows for continuous and independent monitoring as it does not require the installation of anchors systems. However, they are not incompatible monitoring systems in any case, as in recent years, some manufacturers have developed Electronic Performance Tracking Systems (EPTSs), which both integrate sensors and provide a deeper overview of the physical demands [10].

The integration of performance evaluation through globally recognized methodologies and load monitoring using validated devices has driven recent studies exploring the relationship between game performance and physical demands [9,11,12]. Nevertheless, findings to date have been inconsistent to our knowledge. For example, in male basketball players, studies employing traditional statistical metrics and positioning system data have not found significant relationships [11,13]. Conversely, in female basketball players, significant relationships have been reported using inertial sensors and advanced statistical metrics [9]. Espasa et al. (2024) [9] highlighted methodological differences, including data acquisition methods, variables used, and statistical analyses developed, as the primary reasons for these discrepancies. While there is evidence indicating that performance and physical load are related, there is a lack of knowledge about which specific metrics are most significantly associated with the different facets of statistical performance in each playing position. This gap is significant because both constructs are intrinsically linked, and fatigue may impair a player’s ability to execute technical skills and make effective tactical decisions [14]. For these reasons, proper load management is essential to maximize performance without compromising a player’s health [15,16].

Conceptually, this study is based on the premise that a player’s statistical output is a direct result of physical actions, which in turn generate a measurable physical demand. The accumulation of these demands can lead to fatigue, potentially affecting subsequent technical execution and tactical decision-making. Therefore, understanding the physical cost of each game’s action is fundamental. To provide a clear focus, this study aims to answer the following research questions: (1) what is the relationship between specific game performance statistics and physical demands measured by positioning and inertial sensors in youth male basketball players? (2) How do these relationships differ across playing positions (guards, forwards, and centers)?

## 2. Materials and Methods

### 2.1. Participants

Fifteen young and highly trained [17] male basketball players (mean age: 16.3 ± 0.7 years at the start of the season; height: 196.3 ± 11.4; body mass: 88.1 ± 11.4; years of experienced in federative competition: 5.2 ± 1.2) from a 4th Spanish League Team participated in this study (from October 2023 to April 2024). All players participated in at least two games. The players were categorized by coaches as follows: (1) guards; (2) forwards; and (3) centers [5]. The players were categorized by the technical staff based on consensus, indicating which player corresponded to each of the categories. All participants were healthy before starting data collection.

Players were routinely monitored during the entire competitive season [18]. This study was approved by the Ethics Committee of Clinical Research of the Sports Administration of Catalonia (code 013/CEICGC/2022) and the Institutional Review Board of the club. Also followed the ethical standards of the Committee for Responsible Human Experimentation (established by Law 14/2007, Spain) and the principles of the Declaration of Helsinki [19]. Before collecting data, all players were informed about the research procedures and agreed to participate providing written consent. The data obtained were treated with confidentiality, complying with the Organic Law 15/1999 of the 13th of December on the Protection of Personal Data and the General Data Protection Regulation applicable within the European Union [20].

### 2.2. Procedures

**Figure 1:**
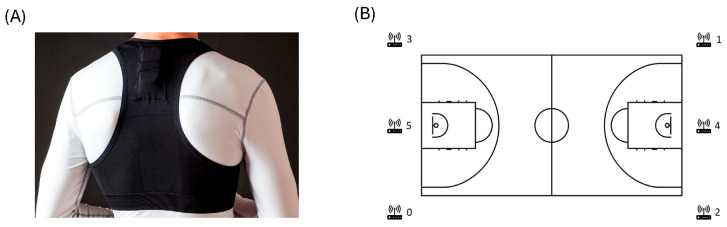
(**A**) Positioning of the device in the upper back and anchors distribution in the court. (**B**) Position of anchors in the local positioning system.

A descriptive and comparative design was used to examine the relationship between player’s physical demands and performance. All games were conducted following FIBA basketball rules during the complete 2023/24 season. Data was collected from a total of seven official games, which were played at the home arena. All players were monitored using performance-tracking devices (Wimu, Hudl^®^ SL, Lincoln, NE, USA). The device (81 × 45 × 15 mm, 70 g) was placed on the upper back using an adjustable top (Figure 1A). This device collects different sensor signals through ultrawide band and inertial technologies. Using ultrawideband signals into a local reference system setup through a total of six anchors (Figure 1B), the device might register during every moment its position in the court and its position changes over time, letting it self-calculate variables related to space and time (speed and accelerations). On the other hand, the device is equipped with a total of four accelerometers that record in different magnitude spectrums (×2: 16 G; ×1: 32 G; ×1: 400 G at 1000 Hz), three gyroscopes (×2: ±2000 degrees per second; ×1: ±4000 degrees per second at 1000 Hz), a magnetometer (±8 Gauss at 160 Hz), and one barometer (±1200 mbar at 100 Hz) using a sampling frequency of 100 Hz [21]. All variables used are described below. All games were video-recorded using automatic cameras, Pixellot Show S3 (Pixellot^®^, Tel Aviv, Israel). The video, positioning, and IMU signals were synchronized using the specific software of the manufacturer, SPRO^TM^ (version 2.2.0, Wimu, Hudl^®^ SL, Lincoln, NE, USA). After this, game periods and player activity based on the court signals were identified and selected for the analysis, excluding intervals between quarters and timeouts. Subsequently, the data collected during these periods were incorporated into the final statistical analysis.

### 2.3. Variables

All variables studied were categorized as independent and dependent. While the metrics used for game performance evaluations were considered independent, the variables related to physical demands were considered dependent, considering two subcategories based on the methods used: positioning or inertial sensors.

#### 2.3.1. Game Performance Variables

Statistical data were retrieved from the official website of the Spanish Basketball Federation (https://baloncestoenvivo.feb.es/, accessed on 28 November 2024) and recorded in a customized Excel^®^ spreadsheet (Microsoft Corporation, Redmond, Washington, DC, USA). The variables used for game performance analysis included the following: (1) total minutes played; (2) points scored; (3) free throws made and attempted (FT: M/A); (4) two-point field goals made and attempted (2P: M/A); (5) three-point field goals made and attempted (3P: M/A); (6) offensive rebounds (OR); (7) defensive rebounds (DR); (8) total rebounds (TR); (9) assists; (10) steals; (11) turnovers; (12) fouls committed; (13) fouls received (FRs); (14) efficiency rating (ER); and (15) balance. From this data, advanced statistical variables were calculated based on possessions and total opportunities in each action. The advanced variables included the following: (1) effective field goal percentage (eFG%); (2) true shooting percentage (TS%); (3) assist–turnover ratio (A/T); (4) offensive rebound percentage (ORB); (5) defensive rebound percentage (DRB); (6) possessions; (7) points per possession (PPP); and (8) player usage percentage (PU%). The formulas used to compute advanced statistics were based on those proposed by the National Basketball Association [5]. Furthermore, player position was treated as a categorical independent variable, classifying players based on their tactical roles on the court. Game quarters and different matches were also considered temporal independent variables, allowing for the examination of how physical demands fluctuate over the course of a game and among various matches.

#### 2.3.2. Physical Demand Variables: Positioning System

The variables derived from the players’ positioning on the court were as follows: (1) total distance covered (TD); (2) absolute high-speed running (HSR: >18 km/h); (3) number of high-intensity accelerations (Hi-Acc: >3 m/s^2^); and (4) number of high-intensity decelerations (Hi-Dec: <-3 m/s^2^). The dwell time of acceleration was fixed at 0.5 s [22].

#### 2.3.3. Physical Demand Variables: Inertial Movement Units

The variables reported by the sensors included in the analysis were as follows: player load (PL), high-intensity player load (Hi-PL, >2G) [23], jumps, jumps’ high-intensity takeoff (Hi-Takeoff), jumps’ high-intensity landing (Hi-Landing) [24], high-intensity horizontal impacts (Hi-HI) [25], the sum of change in inertia (COI), and the sum of high-intensity change in inertia (Hi-COI) [26].

### 2.4. Statistical Analysis

Descriptive (mean ± standard deviation) and normality tests (Shapiro–Wilk) were initially performed for all variables based on two levels, all players and clustered by position, to determine the data distribution for each case. Parametric and non-parametric tests were performed in each case. To assess the correlation among variables, partial correlation analyses were conducted, controlling for the variable of minutes played. These partial correlation analyses were performed for both the team and position grouping. Accordingly, correlation analyses were developed based on player positions. The analysis was adapted to the distribution of the data, using Pearson’s or Spearman’s test (r or Rho, respectively). The strength of the correlation coefficients was interpreted as small (0–0.3), moderate (0.31–0.49), large (0.5–0.69), very large (0.7–0.89), and near perfect (0.91.0) following Cohen’s scale [27]. Statistical significance was set at *p* < 0.05. All data were analyzed using the Jamovi project (2023), Jamovi^®^ (Version 2.3) [28].

## 3. Results

Firstly, to provide context for the data on load and performance during gameplay, the findings from the analyses of various contextual game factors are presented (Table 1).

The results of the descriptive and normality analyses are presented in Table 2, grouping by the team and playing positions. When evaluating the distribution of the data, the independent variables (game performance) that showed a normal distribution were balance, and PU%. In the case of the dependent variables (physical demand variables), on one hand, the normality was met for only for TD, PL, and Hi-PL.

On the other hand, after analyzing the distribution by positions, in the case of guards, the normality was found for minutes played, 2P%, DR, TR, assists, FR, +/−, balance, eFG%, DRB, possessions, PPP, and PU%, while normality was identified for all physical demands’ variables except Hi-HI. Regarding forwards, the variables that showed a normal distribution of their game performance were only minutes, balance, and PPP. For physical demands, all variables followed normality except Hi-HI, jumps, Hi-Takeoff and Hi-Landings. Finally, centers showed normality for minutes, 2PA, +/−, balance, TS%, possessions, PPP, and PU% and for all physical demand’s variables except for Hi-HI.

### 3.1. Physical Demand During Competition by Positions

The descriptive analysis of physical demand data revealed positional differences in movement volume and high-intensity actions (Table 2).

Guards covered the greatest total distance on average (TD = 2965.81 ± 1453.80 m) and recorded the highest number of high-intensity accelerations (Hi-Acc = 39.07 ± 21.16) and decelerations (Hi-Dec = 42.79 ± 26.54), emphasizing their greater movement workload. Forwards displayed moderate values across most metrics, with jumps (24.53 ± 13.29) and player load (PL = 42.69 ± 14.43 AU) standing out as key variables. In contrast, centers recorded the lowest movement-related values (TD = 2207.62 ± 1143.64 m) but exhibited higher values for jumping-related variables, such as Hi-Takeoff (11.00 ± 7.22) and Hi-Landing (11.38 ± 6.91).

Significant differences were observed in high-intensity variables, with guards representing the highest number of Hi-Acc and Hi-Dec actions (*p* < 0.05). In contrast, centers exhibited a significantly greater number of Hi-Landings (*p* < 0.01).

### 3.2. Correlations Between Game Performance and Physical Demands

#### 3.2.1. Partial Correlations by Team Data

The results of the partial correlation analyses between game performance and physical demands are presented in Table 3 and Table 4. The analysis revealed that the strength of these relationships ranged from small to large (0.234–0.604). Notably, large correlations were identified for points, 2PA, ER, TR, PPP, DR, PPM, PU%, 3PA, and 2PM with various physical demand metrics.

When analyzing the correlations between game performance variables and physical demands derived from positioning metrics, the strongest associations were observed between points and TD (r = 0.646, *p* < 0.001), as well as Hi-Acc (Rho = 0.503, *p* < 0.01). In contrast, Hi-Dec demonstrated the strongest correlation with assists (Rho = 0.46, *p* < 0.01). The HSR exhibited only small correlations with various performance metrics, with the most notable relationship observed with TS% (Rho = 0.286, *p* < 0.05). However, the remaining correlations between positioning metrics and game performance variables were statistically significant (*p* < 0.001).

Regarding inertial sensor-derived metrics, 2PA exhibited large correlations with PL (r = 0.626, *p* < 0.001), Hi-Takeoff (Rho = 0.566, *p* < 0.01), Hi-Landing (Rho = 0.549, *p* < 0.01), and COI (Rho = 0.520, *p* < 0.01). Similarly, points scored demonstrated strong correlations with PL (r = 0.603, *p* < 0.001), Hi-PL (Rho = 0.548, *p* < 0.01), jumps (Rho = 0.644, *p* < 0.001), and Hi-Takeoff (Rho = 0.527, *p* < 0.01). In the case of ER, large correlations were found with PL (r = 0.584, *p* < 0.001) and Hi-Landing (Rho = 0.501, *p* < 0.01). TR exhibited large correlations with Hi-Takeoff (Rho = 0.505, *p* < 0.01) and Hi-Landing (Rho = 0.576, *p* < 0.001), while DR showed a significant correlation with Hi-Landing (Rho = 0.546, *p* < 0.01). For PU%, the strongest correlation was found with jumps (Rho = 0.517, *p* < 0.01). Additionally, PPP displayed strong correlations with jumps (Rho = 0.547, *p* < 0.01). Lastly, 2PM was significantly correlated with jumps (Rho = 0.501, *p* < 0.01).

**Table 4:** Partial correlation levels between game performance and physical demands derived from inertial sensors.

Variables	PL (au) ^a^	Hi-PL (au) ^a^	Jumps (*n*) ^β^	Hi-Takeoff (*n*) ^β^	Hi-Landings (*n*) ^β^	Hi-HI (*n*) ^β^	COI (*n*) ^β^	Hi-COI (*n*) ^β^
**Points**	0.605 ***	0.533 ***	0.665 ***	0.562 ***	0.518 ***	0.356 **	0.492 ***	0.349 **
**FTM**	0.417 ***	0.367 **	0.446 ***	0.425 ***	0.400 ***	0.491 ***	0.233	0.229
**FTA**	0.396 ***	0.354 **	0.448 ***	0.443 ***	0.407 ***	0.482 ***	0.218	0.216
**2PM**	0.495 ***	0.456 ***	0.510 ***	0.521 ***	0.448 ***	0.181	0.410 ***	0.226
**2PA**	0.625 ***	0.565 ***	0.650 ***	0.573 ***	0.555 ***	0.178	0.527 ***	0.337 **
**3PM**	0.349 **	0.290 *	0.431 ***	0.205	0.169	0.069	0.240 *	0.177
**3PA**	0.322 **	0.240 *	0.384 ***	0.163	0.118	0.047	0.356 **	0.337 **
**TR (** ***n*** **)**	0.501 ***	0.513 ***	0.493 ***	0.500 ***	0.573 ***	0.289 *	0.412 ***	0.244 *
**OR (** ***n*** **)**	0.313 **	0.308 **	0.322 **	0.321 **	0.359 **	0.160	0.288 *	0.074
**DR (** ***n*** **)**	0.459 ***	0.473 ***	0.485 ***	0.489 ***	0.538 ***	0.270 *	0.392 ***	0.271 *
**Assists (** ***n*** **)**	0.472 ***	0.407 ***	0.327 **	0.249 *	0.312 **	0.122	0.371 **	0.279 *
**Steals (** ***n*** **)**	0.307 **	0.245 *	0.126	0.116	0.130	0.185	0.226	0.157
**TO (** ***n*** **)**	0.412 ***	0.354 **	0.304 **	0.377 **	0.274 *	0.269 *	0.245 *	0.235 *
**Blocks (** ***n*** **)**	0.190	0.221	0.226	0.293 *	0.248 *	0.242 *	−0.013	−0.072
**Dunks (** ***n*** **)**	0.279 *	0.285 *	0.318 **	0.358 **	0.337 **	0.141	0.050	−0.130
**FC (** ***n*** **)**	0.227	0.212	0.325 **	0.250 *	0.216	0.143	0.309 **	0.239 *
**FR (** ***n*** **)**	0.397 ***	0.332 **	0.339 **	0.369 **	0.317 **	0.469 ***	0.307 **	0.285 *
**ER (au)**	0.602 ***	0.559 ***	0.524 ***	0.512 ***	0.519 ***	0.381 **	0.443 ***	0.283 *
**+/− (au)**	0.337 **	0.312 **	0.336 **	0.243 *	0.223	0.192	0.302 *	0.294 *
**Possessions (** ***n*** **)**	−0.034	−0.081	−0.016	−0.076	−0.111	−0.164	−0.002	−0.053
**PPP (au)**	0.464 ***	0.423 ***	0.566 ***	0.471 ***	0.421 ***	0.318 **	0.349 **	0.259 *
**eFG (%)**	0.316 **	0.281 *	0.362 **	0.372 **	0.291 *	0.178	0.220	0.013
**ORB (%)**	0.159	0.182	0.268 *	0.270 *	0.329 **	0.128	0.225	0.035
**DRB (%)**	0.253 *	0.275 *	0.378 **	0.401 ***	0.471 ***	0.228	0.277 *	0.181
**TS (%)**	0.424 ***	0.391 ***	0.391 ***	0.365 **	0.325 **	0.275 *	0.293 *	0.155
**A/TO**	0.270 *	0.205	0.216	0.121	0.177	0.041	0.331 **	0.234 *
**PU (%)**	0.359 **	0.312 **	0.524 ***	0.426 ***	0.387 ***	0.244 *	0.313 **	0.270 *

* *p* < 0.05, ** *p* < 0.01, *** *p* < 0.001; a: Pearson’s correlation coefficients; β: Spearman correlation coefficients. +/−: player balance; 2P: two-point field goals (M: made; A: attempted); 3P: three-point field goals (M: made; A: attempted); A/TO: assists–turnover ratio; COI: changes of inertia; DR: defensive rebounds; DRB: defensive rebound percentage; eFG: efficiency field goal percentage; ER: efficiency rating; FC: fouls committed; FR: fouls received; FT: free throws (M: made; A: attempted); Hi-COI: high-intensity changes of inertia; Hi-HI: high-intensity horizontal impacts; Hi-Landing: jumps’ high-intensity landing; Hi-PL: high-intensity player load; Hi-Takeoff: jumps’ high-intensity takeoff; OF: offensive rebounds; ORB: offensive rebound percentage; PL: player load; PPP: points per possession; TR: total rebounds; USG: usage percentage. AU: arbitrary units. Cell shading corresponds to the magnitude of the correlation, based on the scale by Cohen: light blue (small, 0.01–0.30), medium-light blue (moderate, 0.31–0.49), blue (large, 0.50–0.69).

#### 3.2.2. Partial Correlations by Positions

Partial correlations between performance and physical demand data were analyzed by position. Analyzing the physical demand variables by positions, the distribution of data was normal except in the case of Hi-HI for guards and centers. Table 5 includes the total number and maximal level of correlation of partial correlations among variables from LPS and IMU systems by groups for the rest of them.

When analyzing correlations by playing position, guards exhibited the lowest number of significant associations, both with positioning system variables (*n* = 7) and inertial sensor metrics (*n* = 19). However, all significant partial correlations identified were of large or very large magnitude (0.606–0.791). The strongest correlation among positioning variables and game statistics was observed between HSR and eFG% (r = 0.651, *p* < 0.05). Additionally, there was an inverse correlation between OR and HSR (Rho = −0.616, *p* < 0.05). Among inertial sensor metrics, assists exhibited the highest number of correlations (*n* = 4), though the strongest association was found between 3PM and jumps (Rho = 0.791, *p* < 0.01). The jumps and PL metrics were the most frequently correlated with performance variables.

Forwards exhibited a total of 14 significant correlations between positioning system variables and game performance metrics. Among these, TD showed the highest number of correlations (*n* = 10), with its strongest association observed with steals (Rho = 0.492, *p* < 0.01). Regarding inertial sensor-derived metrics, a total of 42 correlations were identified, which is three times more than those detected for positioning system metrics. Among these, the most notable relationships were as follows: jumps and 2PA (Rho = 0.847, *p* < 0.001); Hi-Takeoff and TR (Rho = 0.573, *p* < 0.01), as well as FR (Rho = 0.592, *p* < 0.01); and assists and Hi-Landing (Rho = 0.356, *p* < 0.05).

Centers exhibited the highest number of significant correlations. The highest correlation values were observed between TD and points scored (Rho = 0.836, *p* < 0.001), 2PA (Rho = 0.815, *p* < 0.001), and assists (Rho = 0.732, *p* < 0.001). Additionally, jumps were strongly correlated with points scored (Rho = 0.863, *p* < 0.001) and 2PA (Rho = 0.847, *p* < 0.001). The Hi-HI variable showed a very large correlation with OR (Rho = 0.794, *p* < 0.001).

## 4. Discussion

The primary objective of this study was to analyze the relationships between game performance and physical demands in male basketball players, utilizing metrics from positioning systems and inertial sensors. The main findings confirmed that game performance metrics are significantly correlated with physical demands, identifying a greater relationship between points and TD and Hi-Acc, while assists showed a moderate level of correlation with Hi-Dec, suggesting that rapid deceleration actions may facilitate playmaking opportunities. Regarding the relationship between performance and the metrics of IMUs, 2PA correlated with PL, Hi-Takeoff, and Hi-Landing, and COI, while points demonstrated significant correlations with PL, Hi-PL, jumps, and Hi-Takeoff, being both the performance variables that more correlated with physical demands. This suggests that higher outputs in game performance metrics could mean that the player was exposed to a higher demand too. This finding could be of interest to better contextualize and understand the physical demands during competition, particularly in the context where monitoring technology is unavailable.

To address the study’s objectives, the authors introduced two methodological modifications compared to prior scientific literature, which are detailed in the following sections. Subsequently, the results obtained from the analyses are discussed.

The first methodological consideration is the treatment of performance metrics as independent variables and physical demands as dependent variables. While this approach is not novel [9], it involves analyzing the data under the assumption that observed physical demands are the result of specific game actions (successful or otherwise). Conversely, the opposing framework has led some authors to claim that physical demands facilitate game performance [11,13,29]. Although both constructs are inseparable and essential for understanding performance and physical demands, it is recommended not to interpret physical demands recorded during gameplay as indicators of fitness levels or athletes’ maximal conditional capabilities. These values are instead a result of the chaotic and stochastic nature of the game, influenced by tactical intentions from coaches, individual player talent, and other factors. An example, based on the findings of this study, is the identification of a relationship where a higher volume of shots (a statistical performance indicator) translates into an increased number of jumps (physical demands), especially accentuated in the center position. The authors consider this a key point for reflection, which could help future researchers and practitioners better understand the relationship between game performance and the physical efforts required.

Moreover, this study employs partial correlation analyses controlling for playing time. This approach, previously proposed in earlier studies [9], differs from the bivariate correlations commonly used in this type of analysis [11,13,30,31,32]. This previous research has proposed two approaches to address the issue of varying playing time exposure in sports with unlimited player substitutions: setting arbitrary participation thresholds or using time-relative metrics. The first approach excludes players who, despite participating for only a few minutes, may play decisive roles in the team’s performance. The second, while potentially addressing the limitations of the former, may not fully account for the cumulative impact of fatigue throughout a game. Additionally, this second type of analysis requires meticulous data handling to exclude continuous pauses during the game, which could otherwise overlook high-intensity actions occurring during stoppages (e.g., baseline or sideline throw-ins).

The use of partial correlations aims not only to mitigate exposure differences among players but also to acknowledge that the initial minutes of play occur under conditions of lower fatigue, which progressively increases as the game unfolds. This factor, not previously emphasized in the literature, significantly influences tactical decisions by coaches and the execution of high-intensity actions by players and should be further explored in future research. While the partial correlation approach may have its own limitations, it is proposed as a starting point for collective reflection in the search for a solution to better understand the impact of it on performance, physical demands, and fatigue.

### 4.1. Performance and Physical Demand Correlations

Our analysis revealed significant associations between game performance and positioning metrics, offering a new perspective compared to previous research. Notably, TD and Hi-Acc demonstrated strong correlations with points scored (r = 0.604, *p* < 0.001; and Rho = 0.526, *p* < 0.001, respectively). This finding compellingly contrasts with studies like García et al. [11], which found no significant correlations in professional male players using traditional bivariate analyses. Discrepancy underscores the importance of our methodological approach; by employing partial correlations to control playing time, we effectively isolated the direct link between physical output and performance, a relationship that might otherwise be obscured.

The importance of these high-intensity actions is further supported indirectly by Miró et al. [12], who observed that U-18 players performed significantly more Hi-Acc and Hi-Dec in quarters they won. Collectively, these results suggest that while a high volume of movement (TD) is linked to creating scoring opportunities—likely by finding open spaces away from defenders [33] and enabling more shot attempts (2PA and 3PA)—the execution of decisive, Hi-Acc is a key factor distinguishing successful periods of play. This aligns with a fast, aggressive playing style that leverages fast breaks and transition opportunities to increase scoring [34]. Furthermore, a significant correlation was identified between Hi-Dec and assists (Rho = 0.476, *p* < 0.001). This suggests that the ability to brake sharply is a critical attribute for playmakers. Tactically, rapid deceleration often forces a defensive reaction, momentarily creating passing lanes. Biomechanically, this action imposes a significant eccentric load on the lower limbs, highlighting a key physical quality for creating playmaking opportunities. This provides empirical support for the idea that sharp changes in pace can disrupt defensive structures and facilitate ball distribution [35], directly linking a specific physical demand to a critical tactical outcome.

On the other hand, HSR showed few significant correlations and generally low magnitudes, with a slightly higher correlation observed with TS%. Overall, the observed correlations between performance variables and positioning metrics are consistent with previous studies [9,12,36]. Unlike other sports, the small dimensions of the basketball court and frequent interruptions in play make peak speeds (long sprints) less common. Instead, short and explosive actions, such as accelerations, decelerations, and changes of direction, become more relevant [36]. At this point, it is worth questioning whether high-intensity positioning variables (Hi-Acc and Hi-Dec) are the most appropriate metrics to describe physical demands in basketball.

Regarding the inertial sensors, our findings indicate that these metrics, particularly those related to jumping, are exceptionally insightful for quantifying the physical demands of performance. The number of jumps, for instance, showed a large correlation with points scored (Rho = 0.665) and two-point attempts (Rho = 0.650). These results align closely with recent findings in professional female players, where an identical partial correlation methodology revealed a very large correlation between jumps and both points (Rho ≈ 0.81) and two-point attempts (Rho ≈ 0.75) [9]. The consistency of this strong link across male and female basketball underscores the universal importance of jumping for offensive production. This robust, cross-gender evidence strongly supports the argument that jump-related metrics provide a more sensitive and representative measure of the physical demands of scoring than positioning systems alone.

The importance of jumping extends beyond just initiating shots. The positive association between metrics like 2PM or PPP and jumping metrics reinforces that repeated explosive efforts contribute directly to offensive success, likely by creating opportunities during drives to the basket, generating second-chance points, or enhancing efficiency in close-range finishes [9,37]. Furthermore, the correlation between PU% and jumps suggests that players with higher involvement in the offense—who inherently perform more decisive actions—also accumulate a greater number of jumps.

Beyond direct scoring actions, our data demonstrates that IMU metrics effectively capture the overall physical cost of performance. Metrics such PL, Hi-Takeoff, and Hi-Landing also correlated strongly with 2PA. This highlights the physical toll of achieving offensive success, which involves not only generating lower-limb power for the jump itself but also absorbing significant impacts upon landing. This connection between movement volume metrics like TD and PL has been noted in prior research [38], but our findings add a layer of mechanical detail specific to basketball actions.

Finally, this relationship between jumping and physical demand is also evident in non-scoring events like rebounding. We observed significant correlations between TR and DR and metrics such as jumps, Hi-Takeoff, and Hi-Landing. This suggests that rebounding involves substantial physical exertion, associating the act of securing the ball with high-velocity efforts and significant landing impacts.

### 4.2. Performance and Physical Demand Correlations by Positions

In male basketball, physical demands differ significantly across playing positions, primarily due to the unique roles and responsibilities associated with each position. The results of the partial correlations for each playing position revealed notable differences in which performance variables were correlated, the number of correlations observed, and their magnitude.

As mentioned below, basketball is a sport that involves numerous actions such as changes of direction, pivoting, physical contests, and jumps, which may not be fully captured by positioning variables, as they often lack significant player displacement yet require considerable physical effort, the main reason for including IMUs as a load-monitoring method. The results of this research not only showed different correlation levels between game performance metrics and physical demands among positions, but also the game performance and physical demand metrics were different, with some being collected through positioning and others through IMUs. These findings suggest that it could be necessary to study physical demands through different variables for each position. All these observed differences will be discussed in detail below.

#### 4.2.1. Guards

Guards exhibited the fewest significant associations with both positioning variables (*n* = 7) and inertial sensor-derived metrics (*n* = 19). However, all recorded correlations were of high or very high magnitude (r = 0.606–0.791), highlighting that while their load patterns may not be broadly related to a wide range of performance indicators, the intensity of these relationships is notable when they occur. Of particular interest is the strong positive correlation between eFG% and HSR. This is noteworthy as high-speed distance covered did not even show moderate correlations in the team-level analysis. This suggests that highly efficient guards tend to execute movements at higher speeds. Fast-break situations, where guards are typically responsible for ball handling, likely elevate speed and create such scenarios [39]. Furthermore, considering that HSR is likely to occur during counterattacks, it can be hypothesized that these counterattacks contribute to an increase in eFG%. Maintaining possession through offensive rebounds slows the pace of play, reducing the need for high-speed transitions. Since second-chance opportunities allow teams to reset their offense, guards are less likely to sprint in fast-break situations.

All inertial sensor metrics that showed associations with game performance were related to PL and jumping actions (jumps, Hi-Takeoff, and Hi-Landing). Regarding PL, the highest correlations were found with defensive rebounds and assists (r = 0.729, *p* < 0.01; and r = 0.712, *p* < 0.01, respectively). The authors hypothesize that these actions are preceded by physical contests and struggles with opponents, which increase PL. On the other hand, jumping actions were more strongly related to shooting metrics. For example, 3PM (Rho = 0.791, *p* < 0.05) and 3PA (Rho = 0.698, *p* < 0.05) demonstrated strong correlations with jumps, suggesting that intense lower-limb muscle activation is required by guards when performing long-range shots. Future studies should analyze this potential correlation.

#### 4.2.2. Forwards

For forwards, 14 significant associations were identified with positioning system metrics, with TD appearing in 10 of them, although none reached a large magnitude. This phenomenon could indicate that the involvement of forwards in both offense and defense requires covering more ground compared to guards or centers (2965 vs. 2608 and 2708 m, respectively). This idea is further supported by correlations between global performance evaluation metrics such as ER, +/−, or PU and TD. It raises the question of whether the role of this position involves accumulating more meters due to increased movement on both sides of the court, an idea previously noted by other authors [13,39]. Among these correlations, the strongest was observed with steals (Rho = 0.492, *p* < 0.05). This could be attributed to the fact that more steels create transition opportunities, allowing them to exploit open spaces to run in fast-break situations.

A remarkable increase in associations was observed when considering IMU metrics (*n* = 42), tripling the number found for positioning variables. This reinforces the idea that inertial sensors provide a more detailed capture of the variety of explosive and contact actions that characterize the hybrid role of forwards. Among these, notable relationships include jumps with 2PA (Rho = 0.847) and Hi-Takeoff with TR and FR, suggesting that explosive takeoffs are common actions for forwards. These actions appear to be associated with mid-range shots, drives to the basket, and rebound attempts, situations in which there might also be a higher likelihood of drawing fouls.

#### 4.2.3. Centers

Centers exhibited the highest number of significant correlations across a wide variety of metrics, including both positioning and inertial systems. This indicates that their statistical performance (points, shoot attempts, assists, rebounds) is strongly linked to the physical load they endure during games. Notably, there were very high correlations between TD and variables such as points, 2PA, and assists. For centers, the volume of movement may be related to their tactical mobility, both offensively (e.g., creating space to receive passes and score) and defensively (e.g., rotations and help defense). Similarly, jumps emerged as a critical factor, showing strong correlations with points (r = 0.863) and 2PA (r = 0.847). This highlights the reliance of centers on these actions for offensive production, whether in post-up plays, “pick and roll” actions, or offensive rebounds leading to second-chance opportunities. This idea is further supported by the correlation between global performance, measured by ER, and Hi-Takeoff (Rho = 0.828, *p* < 0.001). This finding indicates that part of the physical demand accumulated by inside players is found in the quantity and intensity of these vertical component actions.

For the first time, to the best of the authors knowledge, correlations between Hi-HI and player performance were observed, specifically with offensive rebounds. This finding suggests that physical contact, collisions, and body impacts during rebounding battles are critical determinants of centers’ performance [22]. The physicality required in these paint-area duels demands a high degree of impact resistance, underscoring the unique physical demands of this position. The emergence of Hi-HI as a key performance correlate for centers, particularly for offensive rebounds (Rho = 0.794, *p* < 0.001), offers a quantifiable measure of the “battle in the paint” that traditional statistics or even positioning systems fail to capture. This suggests that a center’s effectiveness is not just about vertical jumping ability, but also about the ability to withstand and utilize high-intensity physical contact to establish position. Physiologically, this implies a greater demand for core stability and muscle mass to absorb and exert forces, which has direct implications for position-specific strength and conditioning programs. Also, these findings highlight that inertial sensors could be of greater value than positioning metrics when studying the physical demands of centers.

#### 4.2.4. Differences Among Positions

The differences observed in correlations across positions (the predominance of certain variables over others and their significance levels) underscore the importance of individualizing the monitoring process for physical demands. While the general analysis of partial correlations provides an integrated team view to identify broad trends affecting collective performance, a more detailed position-specific analysis reveals key nuances associated with the unique characteristics and responsibilities of each role. Combining these two perspectives offers a more comprehensive and practical understanding of the impact of physical demands on performance, enabling both collective and individualized strategic interventions to enhance physical preparation and competitive performance.

### 4.3. Positioning and Inertial Systems for Physical Demand Monitoring in Basketball

At this point, it is important to remember that positioning systems were originally developed for outdoor sports like soccer and rugby [6] and were later adapted for indoor games. These sports not only take place in enclosed arenas (requiring the development and implementation of specific monitoring technologies) but also feature markedly different rules, notably smaller playing areas, and unlimited player substitutions. Given these differences, it is necessary to reflect on whether sports like basketball should reconsider how load-monitoring methods have been utilized to describe and evaluate the physical demands of specific actions.

Although the percentage of correlations between positioning and inertial variables was similar, inertial sensors showed higher levels of correlation and greater significance, both in team-wide analyses and when broken down by positions. For example, in the case of 2PA, only TD demonstrated a large correlation among positioning metrics, whereas six variables derived from IMUs showed large correlations. Findings like this highlight the potential of IMUs compared to positioning systems for describing physical demands in basketball. While past studies have already explored the relationship between these two systems [40], future position-specific analyses could help further refine the metrics of interest for each role on the court, enabling a deeper understanding of the impact of physical demands and allowing for the individualization of recovery and training processes.

### 4.4. Limitations and Future Research

Despite the relevance of these findings, several limitations must be considered. First, it is essential to remember that correlation does not imply causation; therefore, our results should be interpreted as exploratory. To move beyond an association and validate these findings, we consider it crucial for future research to incorporate video analysis, synchronizing performance data with physical demand metrics. This approach would also allow for the inclusion of opponent data (e.g., statistics, physical demands, tactical variables), providing essential context that is currently missing.

Building on this, future studies should employ more sophisticated methodological designs. Leveraging the insights gained here to narrow the selection of variables, longitudinal analyses are necessary to establish how the relationship between physical demands and performance evolves throughout a season, accounting for factors like adaptation and cumulative fatigue. Furthermore, while this study used partial correlations, future work should progress towards more complex statistical models. A logical next step would be multiple regression analysis, which could reveal how a combination of the most relevant physical demand variables explains the variance in key performance indicators. An even more advanced approach would involve applying non-linear models to investigate potential threshold effects or points of diminishing returns; for instance, does the positive relationship between distance and scoring efficiency decline after a certain point due to fatigue? Ultimately, these streams of data could be integrated into machine learning algorithms to build truly predictive models of basketball performance.

Finally, a consensus is needed to refine player categorization beyond traditional positions. As is common practice in professional basketball, identifying specific roles within a position (e.g., “stretch-forward” vs. “post-up forward”) is essential [41]. Such an approach would enable a more granular understanding of the specific physical requirements linked to performance, allowing for the creation of highly individualized monitoring and training strategies.

### 4.5. Practical Applications

Understanding the physical demands reflected in statistical performance during basketball games could be highly valuable. The findings of this study offer several direct applications for coaches, strength and conditioning staff, and performance analysts, aiming to optimize training and player load management.

For practitioners without access to monitoring technology, our results confirm that game statistics can serve as a useful proxy for the physical load experienced by players. A high volume of actions recorded in the box score (e.g., shots, rebounds, assists) suggests not only a greater impact on the game but also a higher physical demand, with equal total minutes played. This insight can help contextualize player fatigue and inform decisions regarding in-game rotations. Furthermore, our data provide clear guidance on technology selection. The findings suggest that IMU solutions are particularly effective for monitoring the demands of basketball, as they exhibit stronger and more numerous correlations with performance outcomes compared to LPS. Given their greater accessibility and lower cost, IMUs represent a practical and valuable monitoring tool, making them a priority for many basketball teams.

The primary practical value of this study lies in its contribution to the individualization of load management based on playing position. For instance, the performance of centers is intrinsically linked to vertical actions. Therefore, their load monitoring should prioritize IMU-derived metrics such as the number of jumps and Hi-HI, which were strongly correlated with their offensive production (points, 2PA) and rebounding success (TR, OR). Consequently, their physical preparation should emphasize the development of plyometric power and the strength to withstand physical contact. In contrast, guards’ efficiency is associated with high-speed movements. Monitoring HSR and the frequency of high-Hi-Acc and Hi-Dec is recommended, as these variables relate to greater eFG% and playmaking. Finally, the hybrid role of forwards requires a balanced monitoring approach, considering both their total distance covered and their volume of explosive actions, such as jumps and PL, to gain a comprehensive understanding of their workload.

In summary, these findings empower practitioners to move from generic load monitoring to a more specific, evidence-based strategy. By tailoring training and monitoring practices to the unique demands of each playing position, coaching staff can better optimize player performance and manage fatigue effectively, ultimately contributing to injury risk mitigation.

## 5. Conclusions

This study confirms that game performance and physical demands are intrinsically related in youth male basketball, but it also provides novel insights by revealing the strength of these relationships through a more rigorous analytical approach. By using partial correlations to control playing time, we demonstrated significant, large-magnitude associations between performance metrics and physical outputs—particularly those captured by inertial sensors—that were not identified in prior research using traditional bivariate methods. Specifically, metrics such as TD, PL, and high-intensity actions like jumps and impacts showed strong links to scoring and rebounding. Our findings therefore not only help filter the noise from numerous variables but also establish a clearer, methodologically sounder connection between specific actions and their physical cost.

The practical implications of these findings are twofold. For practitioners with access to monitoring technology, this study highlights the necessity of a combined approach, using both positioning systems and inertial sensors, with variables tailored to each specific playing position to optimize performance and manage player load. For professionals without access to such technology, our results provide a valuable framework for using game statistics as a proxy to understand and contextualize the physical demands experienced by players, enabling more informed technical-tactical decision-making. However, the study’s correlational design means that causation cannot be inferred, and future research is needed to validate these associations. We recommend longitudinal studies that incorporate synchronized video analysis and opposing data to build more comprehensive, and potentially predictive, models of in-game performance. Acknowledging this limitation, our research provides a critical step forward, offering a more nuanced understanding of the physical demands in basketball and emphasizing the indispensable role of inertial sensors in capturing the key actions that define competitive success.

## Figures and Tables

**Table 1:** Contextual data of games and team’s stats.

	Game 1	Game 2	Game 3	Game 4	Game 5	Game 6	Game 7	All Games
**Round (over 30)**	2	5	7	13	16	19	21	-
**Rival ranking (over 16)**	vs 9th	vs 3rd	vs 6th	vs 7th	vs 11th	vs 2nd	vs 11th	-
**Score**	88–63	86–59	73–66	95–80	68–62	75–87	76–67	533–480
**Win/Lose**	Win	Win	Win	Win	Win	Lose	Win	6/7
**FT (M/A; %)**	28/34; 0.82	25/33; 0.76	13/17; 0.76	21/28; 0.75	15/18; 0.83	20/29; 0.69	17/29; 0.59	139/188; 0.74
**2P (M/A; %)**	18/38; 0.47	20/30; 0.66	12/24; 0.5	16/30; 0.53	16/31; 0.52	23/40; 0.58	19/34; 0.56	124/227; 0.55
**3P (M/A; %)**	8/24; 0.33	7/19; 0.37	12/35; 0.34	14/35; 0.4	7/22; 0.32	3/25; 0.12	7/23; 0.30	58/183; 0.32
**TR: OR/DR (** ***n*** **)**	35: 10/25	38: 7/31	36: 9/27	41: 12/29	37: 7/30	31: 10/21	40: 10/30	258: 65/193
**Sum of Assists (** ***n*** **)**	18	13	14	18	15	13	9	100
**Sum of steals (** ***n*** **)**	12	10	8	7	10	10	7	64
**Sum of TO (** ***n*** **)**	16	27	21	12	19	18	17	130
**Sum of blocks (** ***n*** **)**	4	0	4	1	2	3	1	15
**Sum of dunks (** ***n*** **)**	4	0	4	1	2	3	1	15
**Sum of FC (** ***n*** **)**	22	24	18	17	17	32	23	153
**Sum of FR (** ***n*** **)**	27	23	19	22	17	25	28	161
**Sum of ER (au)**	102	93	77	113	78	66	78	607
**Mean of +/− (au)**	10.42	11.25	2.92	6.82	2.50	−5.00	3.75	4.66
**Mean of possessions (** ***n*** **)**	39.38	33.40	38.85	34.95	44.32	44.75	30.70	38.05
**Mean of PPP (au)**	0.18	0.18	0.15	0.19	0.14	0.17	0.20	0.17
**Mean of eFG (%)**	0.38	0.55	0.39	0.48	0.32	0.38	0.42	0.42
**Mean of ORB (%)**	0.06	0.08	0.05	0.08	0.03	0.05	0.07	0.06
**Mean of DRB (%)**	0.11	0.15	0.14	0.14	0.14	0.08	0.19	0.14
**Mean of TS (%)**	0.44	0.66	0.37	0.51	0.40	0.36	0.49	0.46
**Mean of A/TO**	1.03	0.38	0.73	0.77	0.44	0.55	0.40	0.61
**Mean of PU (%)**	0.20	0.18	0.18	0.16	0.17	0.18	0.20	0.18

+/−: player balance; : 2P: two-point field goals (M: made; A: attempted); 3P: three-point field goals (M: made; A: attempted); A/TO: assists–turnover ratio; DR: defensive rebounds; DRB: defensive rebound percentage; eFG: efficiency field goal percentage; ER: efficiency rating; FC: fouls committed; FR: fouls received; FT: free throws (M: made; A: attempted); OR: offensive rebounds; ORB: offensive rebound percentage; PPP: points per possession; TR: total rebounds; PU (%): usage percentage. au: arbitrary units.

**Table 2:** Descriptive (mean ± SD) and normality data by all games and positions.

	All Players (*n* = 72)	Guards (*n* = 14)	Forwards (*n* = 36)	Centers (*n* = 21)
**TD (m)**	2608.89 ± 1142.35	2965.81 ± 1453.80	2708.99 ± 977.78	2207.62 ± 1143.64
**HSR (** ***n*** **) ^2^**	14.92 ± 7.57	11.57 ± 4.38	14.44 ± 5.45	18.19 ± 10.88
**Hi-Acc (** ***n*** **) ^1,2^**	29.03 ± 14.47	39.07 ± 21.16	28.06 ± 11.30	24.10 ± 11.44
**Hi-Dec (** ***n*** **) ^1,2^**	26.00 ± 17.01	42.79 ± 26.54	24.53 ± 10.69	17.48 ± 9.54
**PL (au)**	42.45 ± 17.81	47.20 ± 24.50	42.69 ± 14.43	38.90 ± 18.49
**Hi-PL (au)**	14.51 ± 6.34	15.87 ± 9.26	14.46 ± 4.87	13.62 ± 6.55
**Jumps (** ***n*** **)**	27.94 ± 18.95	35.93 ± 30.89	24.53 ± 13.29	28.67 ± 16.67
**Hi-Takeoff (** ***n*** **)**	10.00 ± 6.76	11.57 ± 9.74	8.75 ± 4.93	11.00 ± 7.22
**Hi-Landing (** ***n*** **)**	11.18 ± 7.61	13.57 ± 10.98	10.06 ± 6.45	11.38 ± 6.91
**Hi-HI (** ***n*** **) ^2^**	5.40 ± 5.82	8.57 ± 9.71	5.31 ± 4.36	3.19 ± 3.43
**COI (** ***n*** **) ^1,2^**	153.00 ± 75.14	204.93 ± 123.13	147.36 ± 44.97	128.90 ± 63.86
**Hi-COI (** ***n*** **) ^1,2^**	33.92 ± 21.34	50.36 ± 35.20	32.75 ± 13.37	25.19 ± 15.02

1: significant difference between guards and forwards; 2: significant difference between guards and centers; COI: changes of inertia; Hi-COI: high-intensity changes of inertia; Hi-HI: high-intensity horizontal impacts; Hi-Landing: jumps’ high-intensity landing; Hi-PL: high-intensity player load; Hi-Takeoff: jumps’ high-intensity takeoff; HSR: high-speed running; PL: player load; TD: distance covered; au: arbitrary units.

**Table 3:** Partial correlation between game performance and physical demands obtained from positioning system.

Variables	TD (m) ^a^	HSR (*n*) ^β^	Hi-Acc ^β^	Hi-Dec ^β^
**Points**	0.604 ***	0.279 *	0.526 ***	0.396 ***
**FTM**	0.386 ***	0.039	0.266 *	0.231
**FTA**	0.338 **	0.042	0.246 *	0.197
**2PM**	0.436 ***	0.252 *	0.367 **	0.223
**2PA**	0.594 ***	0.280 *	0.434 ***	0.308 **
**3PM**	0.421 ***	0.171	0.375 **	0.315 **
**3PA**	0.445 ***	0.186	0.441 ***	0.431 ***
**TR (** ***n*** **)**	0.230 **	0.188	0.308 **	0.305 **
**OR (** ***n*** **)**	0.300	0.229	0.213	0.231
**DR (** ***n*** **)**	0.337 *	0.155	0.298 *	0.283 *
**Assists (** ***n*** **)**	0.499 ***	−0.052	0.346 **	0.476 ***
**Steals (** ***n*** **)**	0.383 ***	−0.188	0.199	0.340 **
**TO (** ***n*** **)**	0.443 ***	−0.034	0.194	0.294 *
**Blocks (** ***n*** **)**	0.079	0.037	0.061	−0.034
**Dunks (** ***n*** **)**	0.198	0.234 *	0.120	0.052
**FC (** ***n*** **)**	0.217	0.155	0.208	0.251 *
**FR (** ***n*** **)**	0.378 **	−0.012	0.337 **	0.351 **
**ER (au)**	0.522 ***	0.127	0.454 ***	0.423 ***
**+/− (au)**	0.333 **	0.259 *	0.351 **	0.261 *
**Possessions (** ***n*** **)**	0.035	−0.135	0.010	0.032
**PPP (au)**	0.449 ***	0.163	0.377 **	0.257 *
**eFG (%)**	0.299 *	0.289 *	0.269 *	0.159
**ORB (%)**	0.068	0.175	0.155	0.168
**DRB (%)**	0.101	0.108	0.175	0.165
**TS (%)**	0.404 ***	0.305 **	0.344 **	0.249 *
**A/TO**	0.311 **	−0.012	0.291 *	0.407 ***
**PU (%)**	0.367 **	0.065	0.292 *	0.250 *

* *p* < 0.05, ** *p* < 0.01, *** *p* < 0.001; a: Pearson’s correlation coefficients; β: Spearman correlation coefficients. +/−: player balance; : 2P: two-point field goals (M: made; A: attempted); 3P: three-point field goals (M: made; A: attempted); A/TO: assists–turnover ratio; DR: defensive rebounds; DRB: defensive rebound percentage; eFG: efficiency field goal percentage; ER: efficiency rating; FC: fouls committed; FR: fouls received; FT: free throws (M: made; A: attempted); Hi-Acc: high accelerations (>3 m/s^2^); Hi-Dec: high decelerations (< -3 m/s^2^); HSR: high-speed running; OF: offensive rebounds; ORB: offensive rebound percentage; PPP: points per possession; TD: total distance; TR: total rebounds; USG: usage percentage. AU: arbitrary units. Cell shading corresponds to the magnitude of the correlation, based on the scale by Cohen: light blue (small, 0.01–0.30), medium-light blue (moderate, 0.31–0.49), blue (large, 0.50–0.69).

**Table 5:** Number of significant correlations and highest level detected between game performance and physical demands from positioning and inertial systems by positions.

Variables	Guards		Forwards		Centers	
	LPS	IMU	LPS	IMU	LPS	IMU
**FTM**	ND	2; Hi-Takeoff ^β^ = 0.703 **	1; TD ^β^ = 0.374 *	7; PL ^β^ = 0.665 ***	3; Hi-Dec ^β^ = 0.600 **	6; Hi-Takeoff ^β^ = 0.649 **
**FTA**	ND	2; Hi-Takeoff ^β^ = 0.67 *	1; TD ^β^ = 0.367 *	6; PL ^β^ = 0.649 ***	1; Hi-Dec ^β^ = 0.461 *	6; Hi-Takeoff ^β^ = 0.605 **
**2PM**	ND	ND	1; TD ^β^ = 0.373 *	1; Hi-Takeoff ^β^ = 0.357 *	4; TD ^β^ = 0.723 ***	8; Jumps ^β^ = 0.749 ***
**2PA**	ND	ND	1; TD ^β^ = 0.404 *	3; Jumps ^β^ = 0.547 ***	4; TD ^a^ = 0.815 ***	7; Jumps ^a^ = 0.847 ***
**3PM**	1; Hi-Acc^β^: 0.572 *	3; Jumps ^β^ = 0.791 **	ND	1; Jumps ^β^ = 0.376 *	2; Hi-Acc ^β^ = 0.543 *	1; Jumps ^β^ = 0.472*
**3PA**	ND	1; Jumps ^β^ = 0.698 **	ND	1; Jumps ^β^ = 0.511 **	2; HSR ^β^ = 0.592 **	1; Hi-COI ^β^ = 0.575 **
**TR (** ***n*** **)**	ND	1; PL ^a^ = 0.645 *	ND	3; Hi-Landing ^β^ = 0.373 *	2; Hi-Dec ^β^ = 0.481 *	6; Hi-Landing ^β^ = 0.847 ***
**OR (** ***n*** **)**	1; HSR ^β^ = −0.616 *	ND	1; Hi-Dec ^β^ = 0.357 *	ND	ND	5; Hi-Landing ^β^ = 0.617 **
**DR (** ***n*** **)**	ND	1; PL ^a^ = 0.729 **	ND	1; COI ^β^ = 0.344 *	2; Hi-Dec ^β^ = 0.516 *	6; Hi-Landing ^β^ = 0.692 ***
**Assists (** ***n*** **)**	ND	4; PL ^a^ = 0.712 **	1; TD ^β^ = 0.373 *	1; Hi-Landing ^β^ = 0.356 *	4; TD ^β^ = 0.644 **	5; Jumps ^β^ = 0.611 **
**Steals (** ***n*** **)**	ND	ND	1; TD ^β^ = 0.492 **	ND	1; TD ^β^ = 0.444 *	1; Jumps ^β^ = 0.461 *
**TO (** ***n*** **)**	ND	ND	ND	2; Hi-Takeoff ^β^ = 0.526 **	1; Hi-Dec ^β^ = 0.472 *	ND
**Blocks (** ***n*** **)**	ND	ND	ND	ND	ND	2; Hi-HI = 0.609 **
**Dunks (** ***n*** **)**	ND	ND	ND	ND	ND	5; Hi-Takeoff = 0.631 **
**FC (** ***n*** **)**	ND	1; Jumps ^β^ = 0.631 *	1; Hi-Dec ^β^ = 0.36 *	ND	1; HSR ^β^ = 0.497 *	3; COI ^β^ = 0.566 **
**FR (** ***n*** **)**	ND	ND	ND	8; PL ^β^ = 0.615 ***	1; Hi-Dec ^β^ = 0.454	6; Hi-Takeoff ^β^ = 0.592 **
**ER (au)**	ND	1; PL ^a^ = 0.634 *	1; TD ^β^ = 0.426 **	3; PL ^β^ = 0.423 *	3; TD ^a^ = 0.700 ***	7; Hi-Takeoff = 0.828 ***
**+/− (au)**	ND	ND	1; HSR ^a^ = 0.399 *	ND	3; TD ^a^ = 0.575 **	7; Jumps = 0.604 **
**Possessions (** ***n*** **)**	ND	ND	ND	ND	ND	ND
**PPP (au)**	ND	1; Jumps ^a^ = 0.616 *	ND	1; Jumps ^β^ = 0.483 **	3; Hi-Acc ^a^ = 0.693 ***	7; Jumps = 0.668 **
**eFG (%)**	2; HSR ^a^ = 0.651 *	ND	ND	ND	1; TD ^β^ = 0.454 *	6; Hi-Takeoff ^β^ = 0.706 ***
**ORB (%)**	ND	ND	1; Hi-Dec^β^ = 0.374 *	ND	ND	2; Hi-HI^β^ = 0.575 **
**DRB (%)**	1; TD ^a^ = 0.643 *	1; PL ^a^ = 0.606 *	ND	ND	ND	3; Hi-HI^β^ = 0.709 ***
**TS (%)**	1; Hi-Acc ^β^ = 0.612 *	1; Jumps ^β^ = 0.667 *	1; TD^β^ = 0.351 *	ND	3; TD^a^ = 0.583**	6; Hi-Takeoff = 0.623 ***
**A/TO**	1; TD ^β^ = 0.555 *	ND	1; TD^β^ = 0.376 *	ND	3; TD^β^ = 0.541*	5; Hi-PL^β^ = 0.513 *
**PU (%)**	ND	ND	1; TD^β^ = 0.412*	4; Jumps^β^ = 0.71***	ND	ND

* *p* < 0.05, ** *p* < 0.01, *** *p* < 0.001; a: Pearson’s correlation coefficients; β: Spearman correlation coefficients. +/−: player balance; A/TO: assists–turnover ratio; COI: changes of inertia; DR: defensive rebounds; DRB: defensive rebound percentage; eFG: efficiency field goal percentage; ER: efficiency rating; FC: fouls committed; FG: field goals (made/attempted); FR: fouls received; Hi-COI: high-intensity changes of inertia; Hi-HI: high-intensity horizontal impacts; Hi-Landing: jumps’ high-intensity landing; Hi-PL: high-intensity player load; Hi-Takeoff: jumps’ high-intensity takeoff; ND: not detected; OF: offensive rebounds; ORB: offensive rebound percentage; PL: player load; PPP: points per possession; TR: total rebounds; USG: usage percentage. AU: arbitrary units; min: minutes. Cell shading corresponds to the magnitude of the correlation, based on the scale by Cohen: light blue (small, 0.01–0.30), medium-light blue (moderate, 0.31–0.49), blue (large, 0.50–0.69), light-dark blue (very large, 0.70–0.89).

## Data Availability

Data is contained within the article.

## Abbreviations

The following abbreviations are used in this manuscript:

+/−	player balance
2PA	two-points attempted
2PM	two-points made
3PA	three-points attempted
3PM	three-points made
A/TO	assists–turnover ratio
AU	arbitrary units
COI	changes of inertia
DR	defensive rebound
DRB	defensive rebound percentage
eFG%	effective field goal percentage
EPTS	electronic performance tracking system
ER	efficiency rating
FC	fouls committed
FTA	free throws attempted
FTM	free throws made
FR	fouls received
Hi-Acc	High accelerations
Hi-COI	high-intensity changes of inertia
Hi-Dec	high decelerations
Hi-HI	high-intensity horizontal impacts
Hi-Landing	jumps’ high-intensity landing
Hi-PL	high-intensity player load
Hi-Takeoff	jumps’ high-intensity takeoff
HSR	high speed running
IMU	Inertial movement unit
LPS	local positioning systems
OR	offensive rebound
ORB	offensive rebound percentage
PL	player load
TD	total distance
TR	total rebounds
TS%	true shooting percentage
PPP	points per possession
PU%	player usage percentage
